# Microbiology of periprosthetic infections following implant-based breast reconstruction surgery: a multicentric retrospective study

**DOI:** 10.3389/fsurg.2024.1477023

**Published:** 2024-10-14

**Authors:** Andrea Vittorio Emanuele Lisa, Flavia Zeneli, Martina Mazzucco, Benedetta Barbieri, Mario Rietjens, Germana Lissidini, Valeriano Vinci, Michele Bartoletti, Alessandra Belati, Davide Bavaro

**Affiliations:** ^1^ Division of Plastic and Reconstructive Surgery, European Institute of Oncology, Milan, Italy; ^2^ Department of Biomedical Sciences, Humanitas University, Pieve Emanuele, Italy; ^3^ Applied Medical-Surgical Sciences, Department of Surgical Sciences, University of Rome “Tor Vergata”, Rome, Italy; ^4^ Division of Plastic and Reconstructive Surgery, University Hospital Polyclinic of Modena, Modena, Italy; ^5^ Division of Plastic and Reconstructive Surgery, IRCCS University Hospital of Bologna Sant Orsola Polyclinic, Bologna, Italy; ^6^ Division of Plastic and Reconstructive Surgery, IRCCS Humanitas Research Hospital, Milan, Italy; ^7^ Division of Breast Surgery, European Institute of Oncology (IEO), Milan, Italy; ^8^ Division of Infectious Diseases, IRCCS Humanitas Research Hospital, Milan, Italy

**Keywords:** breast implant infection, gram positive bacteria, multiresistant bacteria, breast reconstruction, complications

## Abstract

**Introduction:**

Implant-based breast reconstruction (IBR) is the predominant breast reconstruction technique post-mastectomy, with bacterial infections being a significant complication affecting patient recovery and quality of life. The following study aimed to determine the microbiological features of the causative agents responsible for breast implant infections, with more attention paid to the comparative analysis of Gram-positive and Gram-negative bacteria and their presentation.

**Methods:**

We conducted a retrospective analysis of 214 patients who presented with periprosthetic infection and underwent implant removal following implant-based breast reconstruction at Humanitas Research Hospital and Istituto Europeo di Oncologia between January 2018 and March 2024.

**Results:**

The study revealed that Gram-positive bacteria were more prevalent, with *Staphylococcus* species, particularly *Staphylococcus aureus*, being the most isolated pathogen in both institutions (∼39.96%). In contrast, Gram-negative bacteria were less frequent, with a higher proportion of these pathogens being multi-resistant strains. A significant difference was observed (*p* = 0.007), indicating that individuals with normal BMI have a higher prevalence of Gram-positive infections (88.46%), whereas obese and overweight patients had higher proportions of Gram-negative infections (23.53% and 28.89%, respectively). In addition, smoking status was also significantly associated with pathogen distribution (*p* = 0.032), with active and past smokers being related to higher percentages of polymicrobial infections. Furthermore, positive prophylactic MSSA/MRSA swabs were significantly more associated with *Staphylococcus aureus* infections compared to those with negative results (*p* = <0.001).

**Conclusions:**

Gram-positive bacteria, especially *Staphylococcus* species, dominate the microbiological landscape of implant-based breast reconstruction (IBR) infections. Our findings provide insights into this critical issue, facilitating a more precise choice of empiric antibiotic treatment and prevention strategies. This analysis underscores the necessity for prophylactic protocols and therapeutic approaches tailored to the predominant bacterial groups. Further research is needed to explore long-term trends and resistance mechanisms to improve patient management.

## Introduction

Implant-based breast reconstruction (IBR) is the most common method of reconstruction following breast cancer. According to the American Society of Plastic Surgeons, 151,641 breast reconstructions were performed in the United States in 2022. Among these, 117,957 procedures involved using a direct implant or tissue expander placement (1).

Following mastectomy, the majority of patients opt for alloplastic reconstruction instead of autologous tissue reconstruction because of its advantages, which include a short operation time, simplicity of the procedure, a rapid postoperative recovery, no donor site, and little scarring (2).

Bacterial infection is a commonly known and feared complication of implant-based breast reconstruction, with incidence rates ranging from 1% to 43%, according to the clinical setting and the specific procedure employed (3). Breast implant infections can lead to hospitalization, delays in scheduled chemotherapy or radiotherapy, breast deformation due to implant failure, sepsis, or death (4).

Diagnosis is mostly clinical, although a more specific method for identifying antibiotic resistance is bacterial culture with antibiogram using aspirated periprosthetic fluid or tissue samples. The principal infectious organisms encountered in implant-related infections are reported to be Staphylococcus epidermidis, S. aureus, Escherichia coli, Pseudomonas aeruginosa, Propionibacterium, and Corynebacterium. However, Serratia, Enterococcus, Enterobacter, group B Streptococcus, and Morganella have been implicated in the reconstructive population as well (5). Management of breast implant infections typically involves antibiotic therapy and implant removal is often necessary (6).

Antibiotic prophylaxis and empirical treatment should consider the most commonly involved pathogens and regional resistance levels. While there are no established guidelines for empiric antimicrobial therapy, the rationale behind using empiric broad spectrum antibiotics while waiting for culture results is to appropriately cover a broad range of gram-negative and gram-positive including MRSA (7).

This multicentric study retrospectively analyses patients who underwent immediate or two-stage alloplastic breast reconstruction, complicated by periprosthetic infections at two institutions, Humanitas Research Hospital and European Institute of Oncology in Milan. We hypothesize that reviewing the microbiology data obtained from explanted implant-based breast reconstructions would provide a rational basis for antibiotic selection in the future.

Our goals were to describe management of implant infections with broad-spectrum antibiotics, review treatment related adverse events, and report on outcomes of therapy.

## Materials and methods

We conducted a multicentric retrospective cohort study analyzing the prevalence of the causative microorganisms in 214 patients who experienced breast implant reconstruction from January 2018 to March 2024 at two major healthcare institutions: Humanitas Research Hospital and Istituto Europeo di Oncologia. In the time period analysed, there were performed 9,800 breast reconstructions in both centers, and 2% of them required explantation.

Inclusion criteria were defined as patients with a clinical diagnosis of breast implant infection that led to the surgical removal of the implant, history of mastectomy, and implant-based breast reconstruction. Exclusion criteria included patients without complete medical records and those who did not undergo implant removal.

These patients were assessed based on several factors including age, body mass index (BMI), active or past tobacco use, underlying diseases such as diabetes mellitus, and a history of chemotherapy or radiotherapy. Additionally, surgical variables were recorded, including laterality of the surgery, type of mastectomy operation (total, nipple-sparing, skin-sparing), axillary dissection, type of reconstruction (DPI, two-stage), implant characteristics (volume, manufacturer, type, and use of an ADM), and length of drainage and timing of implant removal.

Finally, microbiological data were collected, such as symptoms of infection, culture results, type of antibiotics administered, pre-operative MSSA/MRSA nasal swab positivity.

Out of all the patients that underwent implant removal, we considered infected all the patients who presented post-operative clinical signs suggestive of an infectious event such as fever, or local signs such as the appearance of local erythema, swelling, wound dehiscence, periprosthetic fluid collection, and fluid secretion.

Subsequently, we focused our attention on the microbiological features of the microorganisms isolated from pre-operative and intra-operative cultures, noted for their Gram-staining characteristics and multi-drug resistance profiles.

Microbiological analysis involved identifying the causative organisms from tissue samples and fluid cultures obtained during pre-operative ambulatory evaluations and at the time of explant surgery.

Data were described as numbers and percentages in the case of categorical variables, mean and standard deviation in the case of continuous and approximately Gaussian variables, or median and range otherwise. Adherence to Gaussian distribution was verified with the Shapiro-Wilks test.

Overall demographic, surgical, and complications data were first summarized descriptively. Differences between groups were explored with Chi-square and Fisher Exact test. The significance threshold was set at 0.05. All analyses were carried out using the STATA version 18 program.

## Results

### Demographics, implant details, and surgical techniques

Our population of 214 patients had a median age of 53.9 years (±9.5 years), with a mean BMI of 25.0 (±4.3). Among these patients, 5 (2.34%) had diabetes, and the smoking status was categorized as never (63.08%), ex-smokers (15.42%), and current smokers (21.50%). Radiotherapy and chemotherapy were received by 68 (31.78%) and 88 (41.12%) patients, respectively. The mastectomy techniques performed included total mastectomy (43.46%), nipple-sparing mastectomy (51.40%), and skin-sparing mastectomy (1.87%). Reconstruction types were predominantly with immediate expander placement (64.49%), followed by direct to implant (DTI) (20.09%), exchange expander-prosthesis (13.55%) and other methods (1.87%). Axillary dissection was performed in 86 (40.19%) cases, and prostheses were used in 90 (42.06%) cases. The average duration of drainage was 18.1 days (±7.2), with 47 patients (25.54%) requiring drainage for more than 21 days (Table 1).

**Table 1:** Baseline patient characteristics.

Patients	*N* = 214
Age	53.9 ± 9.5
BMI	25.0 ± 4.3
Diabetes mellitus	5 (2.34%)
Smoke	
Non-smoker	135 (63.08%)
Ex-smoker	33 (15.42%)
Current smoker	45 (21.50%)
Radiotherapy	68 (31.78%)
Chemotherapy	88 (41.12%)
Mastectomy type	
Total	93 (43.46%)
Nipple sparing	110 (51.40%)
Skin reducing	4 (1.87%)
Not reported	7 (3.27%)
Reconstruction type	
Immediate expander placement	138 (64.49%)
Exchange expander-prothesis	29 (13.55%)
Direct prosthesis placement	43 (20.09%)
Other	4 (1.87%)
Axillary dissection performed	86 (40.19%)
Drainage duration (days)	18.1 ± 7.2

### Pathogens distribution

We collected cultural results from both preoperative samples, which included analyses of periprosthetic fluid or tissue samples taken in ambulatory settings, and intraoperative samples, specifically the analysis of the implant following explantation. Out of the 214 patients, preoperative cultures were not performed in 79 cases (36.92%) and negative in 16 cases (7.48%), for a total of 120 preoperative cultural data. Intraoperative cultures instead showed negative results in 35 cases (16.36%) and not performed in 28 cases (13.08%), for a total of 151 intra-operative cultural data available. We categorized the most common culture findings into Gram-positive bacteria, Gram-negative bacteria, mycobacterium, and fungi. Multiple bacterial growths were reported in 6 (2.80%) pre-operative cultures and 14 (6.54%) intra- operative cultures.

The most common isolated pathogens (Table 2) were Gram-positive bacteria in both pre-operatory (45.79%) and intra-operatory (53.74%) cultural exams. The most common isolated Gram-positive bacteria were *Staphylococcus* species (42.06%–46.26%), mostly *Staphylococcus aureus* (34.11%–32.71%), and *Enterococcus* species (2.34%–4.21%). On the other hand, Gram-negative bacteria represented the second most common class of pathogens isolated, with 24 cases pre-operatively (11.21%) and 37 cases intra-operatively (17.29%). The most common Gram-negative bacteria belong to the *Pseudomonas* species (5.14%–9.35%).

**Table 2:** Specific pathogens isolated in both structures.

Pathogen	Preoperatory	Intraoperatory
Gram-positive	99 (45.79%)	115 (53.74%)
*Staphylococcus* species	90 (42.06%)	99 (46.26%)
S. aureus	73 (34.11%)	70 (32.71%)
S. epidermidis	14 (6.54%)	21 (9.81%)
S. lugdunensis	3 (1.40%)	7 (3.27%)
S. haemolyticus	0	5 (2.34%)
*Streptococcus* species	1 (0.47%)	0
*Enterococcus* species	5 (2.34%)	9 (4.21%)
*Propionibacterium* species	1 (0.47%)	1 (0.47%)
*Corynebacterium* species	1 (0.47%)	1 (0.47%)
Others	1 (0.47%)	5 (2.34%)
Gram-negative	24 (11.21%)	37 (17.29%)
*Pseudomonas* species	11 (5.14%)	20 (9.35%)
*Serratia* species	1 (0.47%)	3 (1.40%)
*Enterobacter* species	3 (1.40%)	3 (1.40%)
*Acinetobacter* species	1 (0.47%)	2 (0.93%)
*Proteus* species	3 (1.40%)	5 (2.34%)
*Escherichia* coli	2 (0.93%)	3 (1.40%)
*Klebsiella* species	2 (0.93%)	1 (0.47%)
*Morganella* species	1 (0.47%)	0
Mycobacterium	0	1 (0.47%)
Fungi	0	1 (0.47%)
*Aspergillus* species	0	1 (0.47%)
Multiple bacteria	6 (2.80%)	14 (6.54%)

We also analyzed the frequency of the different pathogens between the two institutions separately (Table 3).

**Table 3:** Specific pathogens isolated in each structure (IEO & ICH).

	IEO pre	IEO intra	ICH pre	ICH intra	*p*-value
Gram-positive	76 (90.48%)	39 (75.00%)	67 (78.82%)	105 (80.77%)	**0**.**0397**
*Staphylococcus* species	38 (45.24%)	19 (36.54%)	59 (69.41%)	92 (70.77%)	0.6530
S. aureus	34 (40.48%)	17 (32.69%)	39 (45.88%)	53 (40.77%)	0.5505
S. epidermidis	3 (3.57%)	2 (3.85%)	11 (12.94%)	19 (14.62%)	0.2037
S. lugdunensis	1 (1.19%)	0	2 (2.35%)	8 (6.15%)	0.2101
S. haemolyticus	0	0	0	6 (4.62%)	**0**.**0442**
Other Staph	0	0	4 (4.71%)	6 (4.62%)	0.2093
*Streptococcus* species	0	0	1 (1.18%)	0	
*Enterococcus* species	0	1 (1.92%)	5 (5.88%)	8 (6.15%)	0.2484
*Propionibacterium* species	0	0	1 (1.18%)	1 (0.77%)	
*Corynebacterium* species	0	0	0	1 (0.77%)	
Others	0	0	1 (1.18%)	3 (2.31%)	0.5222
Gram-negative	8 (9.52%)	13 (25.00%)	16 (18.82%)	25 (19.23%)	0.1662
*Pseudomonas* species	5 (5.95%)	9 (17.31%)	6 (7.06%)	11 (8.46%)	**0**.**0452**
*Serratia* species	0	1 (1.92%)	1 (1.18%)	2 (1.54%)	0.7067
*Enterobacter* species	1 (1.19%)	1 (1.92%)	2 (2.35%)	2 (1.54%)	0.9493
*Acinetobacter* species	0	1 (1.92%)	1 (1.18%)	2 (1.54%)	0.7067
*Proteus* species	2 (2.38%)	1 (1.92%)	1 (1.18%)	4 (3.08%)	0.8427
*Escherichia coli*	0	0	2 (2.35%)	3 (2.31%)	0.5178
*Klebsiella* species	0	0	2 (2.35%)	1 (0.77%)	
*Morganella* species	0	0	1 (1.18%)	0	
Mycobacterium	0	0	1 (1.18%)	1 (0.77%)	
Fungi	0	0	1 (1.18%)	1 (0.77%)	
*Aspergillus* species	0	0	1 (1.18%)	1 (0.77%)	
Mul1tiple bacteria	0	0	6 (7.06%)	14 (10.77%)	**0**.**0202**

Total results of Gram positive species were significantly higher in ICH (*p* = 0.0397). *Pseudomonas* species resulted significantly more frequent in IEO than in ICH (*p* = 0.0452).

*S. haemolyticus* and bacterial infections caused by multiple pathogens were only found in ICH (*p* = 0.0442 and 0.0202, respectively).

Statistically significant results (*p* ≤ 0.05) are signed in bold.

The analysis of pathogen frequencies between the IEO (Istituto Europeo di Oncologia) and ICH (Istituto Clinico Humanitas) institutions revealed homogeneity in the distribution of Gram-positive and Gram-negative bacteria across preoperative and intraoperative settings.

For IEO, Gram-positive bacteria constituted 90.48% of the pathogens in preoperative samples and 75.00% in intraoperative samples. *Staphylococcus* species were notably prevalent, with *S. aureus* being the dominant pathogen, representing 40.48% of preoperative and 32.69% of intraoperative samples. Other *Staphylococcus* species such as S*. epidermidis* and *S. lugdunensis* were also present but to a lesser extent. Gram-negative bacteria accounted for 9.52% and 25.00% of the pathogens in preoperative and intraoperative samples, respectively. *Pseudomonas* species were the most frequent Gram-negative bacteria, comprising 5.95% and 17.31% of the preoperative and intraoperative samples, resulting significantly more frequent in IEO than in ICH (*p* = 0.0452).

ICH also displayed a high prevalence of Gram-positive bacteria, with 78.82% in preoperative and 80.77% in intraoperative samples. *Staphylococcus* species, particularly *S. aureus*, were again the most common, representing 69.41% of preoperative and 40.77% of intraoperative samples. *Streptococcus* and *Corynebacterium* species were present in small amounts, while *Enterococcus* species appeared more frequently in ICH than in IEO, comprising 5.88% of preoperative and 6.15% of intraoperative samples. Gram-negative bacteria were less dominant in ICH, accounting for 18.82% and 19.23% of the preoperative and intraoperative samples, respectively. *Pseudomonas* species were again the most prevalent Gram-negative bacteria, representing 7.06% of preoperative and 8.46% of intraoperative samples. Other Gram-negative pathogens such as *Serratia*, *Enterobacter*, and *Acinetobacter* species were also present but with lower frequencies. Statistically significant differences were found regarding *S. haemolyticus* and bacterial infections caused by multiple pathogens, which were only found in ICH (*p* = 0.0442 and 0.0202, respectively).

### Distribution of pathogen classes across different patient characteristics

We examined the distribution of different pathogen classes (Gram-negative bacteria, Gram-positive bacteria, mixed infections, fungi, and mycobacteria) across various patient characteristics, including age, BMI, smoking status, past radiotherapy and chemotherapy, axillary dissection, reconstruction type, drainage duration, and timing of implant removal (Table 4).

**Table 4:** Distribution of pathogen classes across different patient characteristics.

Type	Gram-negative	Gram-positive	Mixed	Fungi	Mycobacteria	*p*-value
Age groups						0.728
≤45	6 (20.00%)	22 (73.33%)	2 (6.67%)	0	0	
45–60	17 (15.74%)	84 (77.78%)	6 (5.56%)	0	1 (0.93%)	
≥60	6 (14.29%)	34 (80.95%)	1 (2.38%)	1 (2.38%)	0	
BMI groups						**0** **.** **007**
Normal	4 (5.13%)	69 (88.46%)	4 (5.13%)	1 (1.28%)	0	
Obese	12 (23.53%)	39 (76.47%)	0	0	0	
Overweight	13 (28.89%)	26 (57.78%)	5 (11.11%)	0	1 (2.22%)	
Underweight	0	6 (100.00%)	0	0	0	
Smoking groups						**0**.**032**
Active	4 (10.81%)	29 (78.38%)	3 (8.11%)	1 (2.70%)	0	
Past	5 (16.13%)	21 (67.74%)	4 (12.90%)	0	1 (3.23%)	
No	20 (18.02%)	89 (80.18%)	2 (1.80%)	0	0	
Radiotherapy						0.571
Yes	10 (17.86%)	42 (75.00%)	3 (5.36%)	1 (1.79%)	0	
No	19 (15.32%)	98 (79.03%)	6 (4.38%)	0	1 (0.81%)	
Chemotherapy						0.421
Yes	15 (20.27%)	54 (72.97%)	5 (6.76%)	0	0	
No	14 (13.21%)	86 (81.13%)	4 (3.77%)	1 (0.94%)	1 (0.94%)	
Axillary dissection						0.634
Yes	15 (19.48%)	58 (75.32%)	4 (5.19%)	0	0	
No	14 (13.08%)	82 (76.64%)	5 (4.67%)	1 (0.93%)	1 (0.93%)	
Reconstruction type						**0**.**019**
Two-stage	5 (21.74%)	14 (60.87%)	3 (13.04%)	1 (4.35%)	0	
DTI	24 (15.38%)	125 (80.13%)	6 (3.85%)	0	1 (0.64%)	
Drainage						0.623
≤21 days	23 (19.01%)	90 (74.38%)	6 (4.96%)	1 (0.83%)	1 (0.83%)	
>21 days	3 (6.25%)	42 (87.50%)	3 (6.25%)	0	0	
In site	2 (25.00%)	6 (75.00%)	0	0	0	
Explant timing						0.559
≤30 days	3 (25.00%)	9 (75.00%)	0	0	0	
1–3 months	17 (17.89%)	73 (76.84%)	4 (4.21%)	0	1 (1.05%)	
>3 months	8 (11.11%)	58 (80.56%)	5 (6.94%)	1 (1.39%)	0	

BMI groups and smoking status play a significant role in the type of pathogens that patients are more likely to develop (respectively *p* = 0.007 and *p* = 0.032). Also the type of reconstruction was significantly associated with the type of pathogens (*p* = 0.019).

Statistically significant results (*p* ≤ 0.05) are signed in bold.

Regarding the BMI, we divided our patients in four subgroups according with the World Health Organization classification (Table 5).

**Table 5:** BMI categories according to WHO classification.

BMI range	Group
≤18.5	Underweight
18.5–25	Normal weight
25–30	Overweight
≥30	Obese

Moreover, the timing of implant removal was considered from the day of the reconstruction surgery until the day of the explantation. Reconstruction types include the direct-to-implant (DTI) subgroup, which refers to patients that underwent mastectomy followed by either expander or direct prosthesis placement, and the two-stage reconstruction subgroup, which refers to the substitution of the expander with a definitive prosthesis.

There was no significant difference in the distribution of pathogens among age groups. Among BMI groups, a significant difference was observed (*p* = 0.007), indicating that BMI plays a crucial role in the type of pathogens patients are more likely to develop. Normal-weight patients showed a high prevalence of Gram-positive infections (88.46%), whereas obese and overweight patients had higher proportions of Gram- negative infections (23.53% and 28.89%, respectively). Smoking status was also significantly associated with pathogen distribution (*p* = 0.032). Non-smokers had a higher proportion of Gram-negative infections (18.02%), while past smokers and current smokers exhibited higher percentages of mixed infections (8.11% and 12.90%, respectively). Radiotherapy and chemotherapy did not show significant associations with pathogen types. The analysis of axillary dissection showed no significant correlation with pathogen distribution. However, the type of reconstruction was significantly associated with the type of pathogens (*p* = 0.019). Patients undergoing two-stage reconstruction had higher rates of mixed infections (13.04%) compared to those undergoing direct-to-implant (DTI) reconstruction, who showed a higher prevalence of Gram-positive infections (80.13%).

Finally, drainage duration and timing of implant removal did not show significant difference in the type of pathogens found.

### Concordance of preoperative and intraoperative cultures

Among the 214 patients, cultures resulted positive both preoperatively and intraoperatively in 72 cases. Concordance between the pathogens found in preoperative and intraoperative culture results was observed (Table 6), with 56 cases (24.30%) showing concordant results, 6 cases showing discordant results, and 10 cases (4.67%) partially concordant (in cases of infections caused by multiple microorganisms, one pathogen was consistently identified in both preoperative and intraoperative culture results, while additional pathogens were newly detected in the intraoperative samples).

**Table 6:** Concordance between preoperatory and intraoperatory culture results.

Total	
Concordant	56 (77.78%)
Discordant	6 (8.33%)
Partially concordant	10 (13.89%)

### Time of onset and pathogen distribution

This study also examined the onset based on the pathogens involved in implant infections (Table 7). The onset of infections was categorized as either early (within 6 weeks from implant or expander positioning) or late (more than 6 weeks). We also evaluated the antibiotic resistance profiles of the microorganisms. Those found to be resistant to three or more antibiotics on the antibiogram were classified as multi- resistant.

**Table 7:** Time of onset and pathogen distribution.

Pathogens	Early onset	Late onset	*p*-value
Total cases	133	81	
With cultural data available	129 (96.99%)	77 (95.06%)	0.481
Gram-positive	100 (77.52%)	60 (77.92%)	0.946
Multi-resistant bacteria	61/95 (64.21%)	35 (58.33%)	0.500
*Staphylococcus* species	59 (59.00%)	29 (48.33%)	0.195
*Staphylococcus* aureus	51 (51.00%)	22 (36.67%)	0.101
*Streptococcus* species	1 (1.00%)	0	
*Enterococcus* species	5 (5.00%)	0	
*Propionibacterium* species	1 (1.00%)	0	
*Corynebacterium* species	1 (1.00%)	0	
Gram-negative	32 (24.81%)	11 (14.29%)	0.946
Multi-resistant bacteria	24 (75.00%)	10/10 (100%)	0.165
*Pseudomonas*	9 (28.12%)	2 (18.18%)	0.698
*Serratia*	1 (3.12%)	0	
*Enterobacter*	1 (3.12%)	2 (18.18%)	
*Acinetobacter*	1 (3.12%)	0	
*Proteus*	2 (6.25%)	1 (9.09%)	
*Escherichia coli*	2 (6.25%)	0	
*Klebsiella*	1 (3.12%)	1 (9.09%)	

Among the 214 patients, 133 experienced early infections, which constituted 62.15% of the cases. For these early cases, cultural data was available for 96.99%, with Gram- positive bacteria being predominant in 77.52% of these cases. Notably, multiresistant bacteria accounted for 64.21% of the Gram-positive infections. *Staphylococcus aureus* was the most frequent pathogen isolated, found in 51.00% of the early cases. Gram- negative bacteria were identified in 24.81% of early infections, with 75.00% being multiresistant and *Pseudomonas* species being the most prevalent at 28.12%.

In contrast, the smaller cohort of 81 patients experiencing late infections accounted for 37.85% of total cases. The distribution of pathogens in these cases mirrored that of the early infections, with Gram-positive bacteria again more common at 77.92% and *Staphylococcus aureus* present in 48.33% of cases. Gram-negative bacteria were less frequent in late infections at 14.29%, and all identified were multiresistant. Statistical analysis revealed no significant differences in the prevalence of mu bacteria among Gram-negative infections between early and late infections (*p* = 0.165), nor in the proportions of Gram-positive or Gram- negative bacteria between the two groups (*p* = 0.946 for both).

### Clinical manifestation

We analyzed the distribution of the different causative agents based on the clinical manifestation (Table 8). Localized symptoms were defined as the presence of breast erythema, pain, calor (increased warmth), fluid collection, secretion, or wound dehiscence. Systemic symptoms were defined by the presence of pyrexia >38°C. We categorized our 214 patients based on their clinical presentations into two distinct groups: those exhibiting only localized symptoms and those showing both localized and systemic symptoms.

**Table 8:** Distribution of pathogens based on clinical manifestation.

Clinical manifestation	Only local	Local and systemic	*p* value
Gram-positive	79 (72.48%)	61 (85.92%)	0.053
Gram-negative	23 (21.10%)	6 (8.45%)	**0**.**040**
Mixed	5 (4.59%)	4 (5.63%)	
Fungi	1 (0.92%)	0	
Mycobacterium	1 (0.92%)	0	

The clinical presentation Gram-negative bacteria infections shows a statistically signifcant difference (*p* = 0.040), indicating that these bacteria are more commonly associated with presenting only local symptoms.

Statistically significant results (*p* ≤ 0.05) are signed in bold.

Among the patients with positive cultural results that presented with localized symptoms, 79 cases were caused by Gram-positive bacteria (72.48%), while Gram- negative bacteria were found in 21.10% of cases. The distribution of Gram-negative bacteria shows a statistically significant difference (*p* = 0.040), indicating that these bacteria are more commonly associated with presenting only local symptoms.

### Antibiotic treatment

The analysis of antibiotic treatment regimens revealed several key insights (Table 9). Of the 214 infected patients, 35 didn't receive any empiric treatment, while 119 did not receive targeted treatment (antibiotic treatment based on culture antibiogram results). The most used empiric antibiotics were sulphonamides, particularly Trimethoprim/sulfamethoxazole, used in 71 cases (33.18%), followed by penicillins, particularly Amoxicillin/clavulanic acid, used in 52 cases (24.30%). In some instances, no antibiotic treatment was administered (16.36%), while antibiotic combinations were used in 3.27% of cases.

**Table 9:** Frequency of antibiotic treatment choice.

Empiric antibiotic	Times it was used	Target antibiotic	Times it was used
Sulphonamides	71 (33.18%)	No antibiotic	119 (55.61%)
Penicillin	52 (24.30%)	Sulphonamides	27 (12.62%)
Cephalosporins	19 (8.88%)	Penicillins	18 (8.41%)
Fluoroquinolones	18 (8.41%)	Multiple antibiotics	18 (8.41%)
Macrolides	10 (4.67%)	Cephalosporin	6 (2.80%)
Multiple antibiotics	7 (3.27%)	Macrolides	3 (1.40%)
Tetracycline	1 (0.47%)	Oxazolidinones	2 (0.93%)
Carbapenem	1 (0.47%)	Lipopeptides	1 (0.47%)

This study also evaluated the effectiveness of antibiotic regimens in achieving culture negativization in breast implant infections (Tables 10, 11), excluding cases where no therapy was administered (35 empiric and 119 targeted therapy cases). We included only cases with positive preoperative cultures, along with intraoperative results that were either positive or negative, to evaluate the efficacy of the antibiotic treatment in achieving negative culture outcomes.

**Table 10:** Empiric antibiotic therapy (excluding untreated cases).

Antibiotic	Positive intraoperative	Negative intraoperative	*P* value
Total cases	72	27	
Sulphonamides	29 (40.28%)	8 (29.63%)	0.458
Penicillin	23 (31.94%)	7 (25.93%)	0.738
Cephalosporins	6 (8.33%)	2 (7.41%)	
Fluoroquinolones	5 (6.94%)	6 (22.22%)	0.073
Macrolides	2 (2.78%)	3 (11.11%)	0.242
Multiple antibiotics	4 (5.56%)	0	0.498
Tetracycline	0	1 (3.70%)	0.608
Carbapenem	1 (1.39%)	0	

**Table 11:** Targeted antibiotic therapy (excluding untreated cases).

Antibiotic	Positive intraoperative	Negative intraoperative	*P* value
Total cases	46	15	
Sulphonamides	15 (32.61%)	1 (6.67%)	0.100
Fluoroquinolones	5 (10.87%)	7 (46.67%)	**0**.**008**
Penicillins	11 (23.91%)	0	0.088
Multiple antibiotics	11 (23.91%)	4 (26.67%)	
Cephalosporin	1 (2.17%)	0	
Macrolides	2 (4.35%)	1 (6.67%)	
Oxazolidinones	1 (2.17%)	1 (6.67%)	
Lipopeptides	0	1 (6.67%)	

Target therapy with fluoroquinolone antibiotics has significantly reduced the presence of bacteria in the intraoperative cultures (*p* = 0.008).

Statistically significant results (*p* ≤ 0.05) are signed in bold.

### Preoperatory MSSA swab

We also analyzed the positivity/negativity of the prophylactic preoperatory MSSA nasal/pharynx swabs (Table 12). Among the 214 patients examined, 68 did not undergo MSSA testing, 111 tests resulted in negative, and 35 resulted in positive.

**Table 12:** MSSA swab results.

MSSA swab	Positive		Negative		*p*-value
Cultural exam	Pre-op	Intra-op	Pre-op	Intra-op	
N	35	35	111	111	
Gram-positive	20 (57.14%)	27 (77.14%)	47 (42.34%)	53 (47.75%)	**0** **.** **0013**
*Staphylococcus aureus*	18 (51.43%)	21 (60.00%)	37 (33.33%)	26 (23.42%)	**<0**.**001**
*Streptococcus* species	0	0	0	0	
*Enterococcus* species	1	1	2	7	
Other	0	0	1	2	
Gram-negative	2 (5.71%)	2 (5.71%)	13 (11.71%)	21 (18.92%)	**0**.**0374**
*Pseudomonas*	1	1	6	13	
*Serratia*	0	0	1	0	
*Enterobacter*	0	0	1	2	
*Acinetobacter*	0	0	1	1	
*Proteus*	0	0	2	3	
*Escherichia coli*	1	0	1	1	
*Klebsiella*	1	0	1	1	
Other	0	1	0	0	

Gram-positive and *Staphylococcus aureus* infections were significantly higher in patients with positive MSSA swabs compared to those with negative results (*p*-values of 0.0013 and <0.0001, respectively). Conversely, Gram-negative infections were significantly lower in the positive MSSA group, with a *p*-value of 0.0374.

Statistically significant results (*p* ≤ 0.05) are signed in bold.

In our analysis, we mostly focused on the distribution of Gram-positive, Staphylococcus aureus, and Gram-negative infections in relation to positive and negative MSSA nasal swab results. The data demonstrated that the proportions of Gram-positive and Staphylococcus aureus infections were significantly higher in patients with positive MSSA swabs compared to those with negative results, with *p*- values of 0.0013 and <0.0001, respectively. Conversely, the proportion of Gram- negative infections was significantly lower in the positive MSSA group, with a *p*-value of 0.0374. These findings indicate a strong association between positive MSSA nasal swab results and increased prevalence of Staphylococcus aureus infections, highlighting the predictive value of MSSA screening in assessing infection risks.

## Discussion

Periprosthetic breast infections usually manifest in a bimodal fashion. Most commonly, early infections present between the first and sixth week after surgery with both local and systemic signs of infection, such as fever, breast pain, erythema, and purulent fluid or drainage at the incision site. Late infections, instead, are less common and mainly present with focal symptoms like dehiscence, drainage, cellulitis, and extrusion of the implant (3).

According to the literature, the most common causative pathogens are coagulase- negative staphylococci, *Staphylococcus aureus*, *Streptococcus pyrogens*, *Propionibacterium acnes*, and *Bacillus* species. Some atypical species like *Klebsiella pneumoniae*, *Clostridium perfringens*, *Serratia marcescens*, and a few fungi may also be present (6).

Managing breast implant infections typically involves antibiotic therapy and, in most cases, implant removal. Antibiotic prophylaxis and empirical treatment should consider regional resistance levels. While there are no established guidelines for empiric antimicrobial therapy, literature recommends that Daptomycin be included in the initial treatment due to the high incidence of beta-lactam-resistant pathogens. Additional coverage for Gram-negative bacteria with broad-spectrum cephalosporins or extended-spectrum penicillins may be appropriate while waiting for culture results.

This study provides an analysis of the microbiological characteristics associated with periprosthetic infections following implant-based breast reconstruction at two major breast cancer centers in Italy. While the existing literature has extensively covered risk factors and treatments for breast implant infections, is a notable lack of research on how epidemiological and microbiological knowledge of these pathogens can facilitate the prompt recognition and treatment of this serious complication, ultimately aiming to preserve implants rather than resort to explantation.

Our study addresses this gap by focusing on the microbiological aspects related to patient characteristics and medical history within the context of breast reconstruction. The insights provided by our research will guide the selection of targeted antibiotic therapies prior to the availability of individual antibiograms. Additionally, our findings aim to establish a protocol that improves the prevention and empiric antibiotic selection based on the suspected pathogen.

With a sample size of 214 patients, we meticulously reviewed and analyzed the medical records, surgical details, and follow-up data of these individuals over a period of 5 years at Humanitas Research Hospital (ICH) and Istituto Europeo di Oncologia (IEO). By comparing the different causative pathogens, we found significant differences in BMI groups, smoking status, reconstruction type, and clinical manifestation.

The findings of our study reveal the most common pathogens found in preoperative and intraoperative cultures. The results indicate that Gram-positive bacteria, particularly *Staphylococcus* species, are the most common pathogens identified both preoperatively and intraoperatively. This aligns with existing literature that underscores the prevalence of *Staphylococcus aureus* in surgical site infections in the field of implant-based breast reconstruction (8–13). Gram-negative bacteria were less frequently identified, but still represented a significant portion of the pathogens, with *Pseudomonas* species being the most prevalent Gram-negative bacteria found, coherently to what has been said in literature (9). This highlights the need for empiric antimicrobial therapy that includes coverage for healthcare associated Gram-negative organisms, particularly *Pseudomonas*. The detection of non-tuberculous *Mycobacterium* in intraoperative cultures, albeit in small numbers, suggests that these pathogens, even if rare, can complicate postoperative outcomes. Periprosthetic mycobacterial infections are often complicated by a delay in diagnosis and can be difficult to manage. Therefore, timely and appropriate bacterial cultures and workup are essential to guide appropriate management (9, 10). The presence of *Aspergillus* species underlines the importance of monitoring for fungal infections, especially in immunocompromised patients or those with a late presentation of symptoms (6).

**Figure 1:**
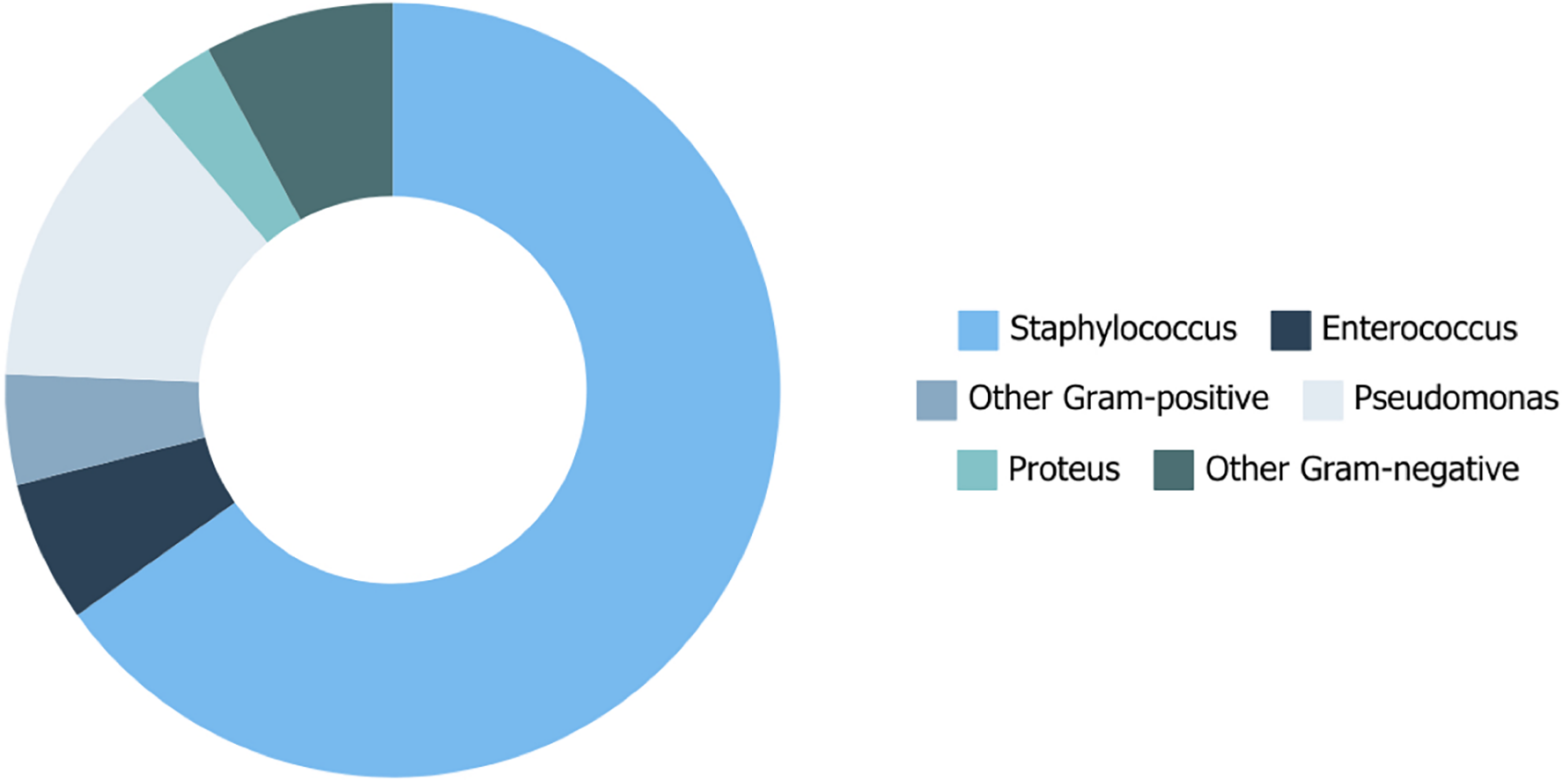
Epidemiologic occurrence rates of different pathogens based on cultural data.

Comparing the epidemiologic occurrence rates of different pathogens between the cultural data from Humanitas Research Hospital (ICH) and Istituto Europeo di Oncologia (IEO) confirmed the homogeneity of the microbiological populations at both institutions. Gram-positive bacteria were the most frequent cause of infection, with *Staphylococcus aureus* accounting for 36.59% of cases at IEO and 55.09% at ICH (Figure 1). The significant presence of Gram-negative bacteria, especially *Pseudomonas* species in IEO, adds an additional layer of complexity to the management of these infections. ICH showed a higher diversity and frequency of Gram-positive pathogens compared to IEO. The high concordance between preoperative and intraoperative cultures for Gram-positive infections supports the use of preoperative cultures to guide initial antibiotic therapy, helping in the early identification of causative pathogens and selection of appropriate antibiotics, thereby increasing the likelihood of successful treatment and salvage of breast implants. In our study, 23.83% of tissue, fluid, and implant cultures yielded negative results. Several factors may account for these negative cultures, such as the use of antimicrobial agents before sample collection, bacterial biofilm formation on the implant surface, the presence of fastidious or slow- growing microorganisms such as *Mycobacterium* species, and infections caused by bacteria like *Cutibacterium* or *Corynebacterium*, which might be mistakenly dismissed as contaminants (10, 11).

**Figure 2:**
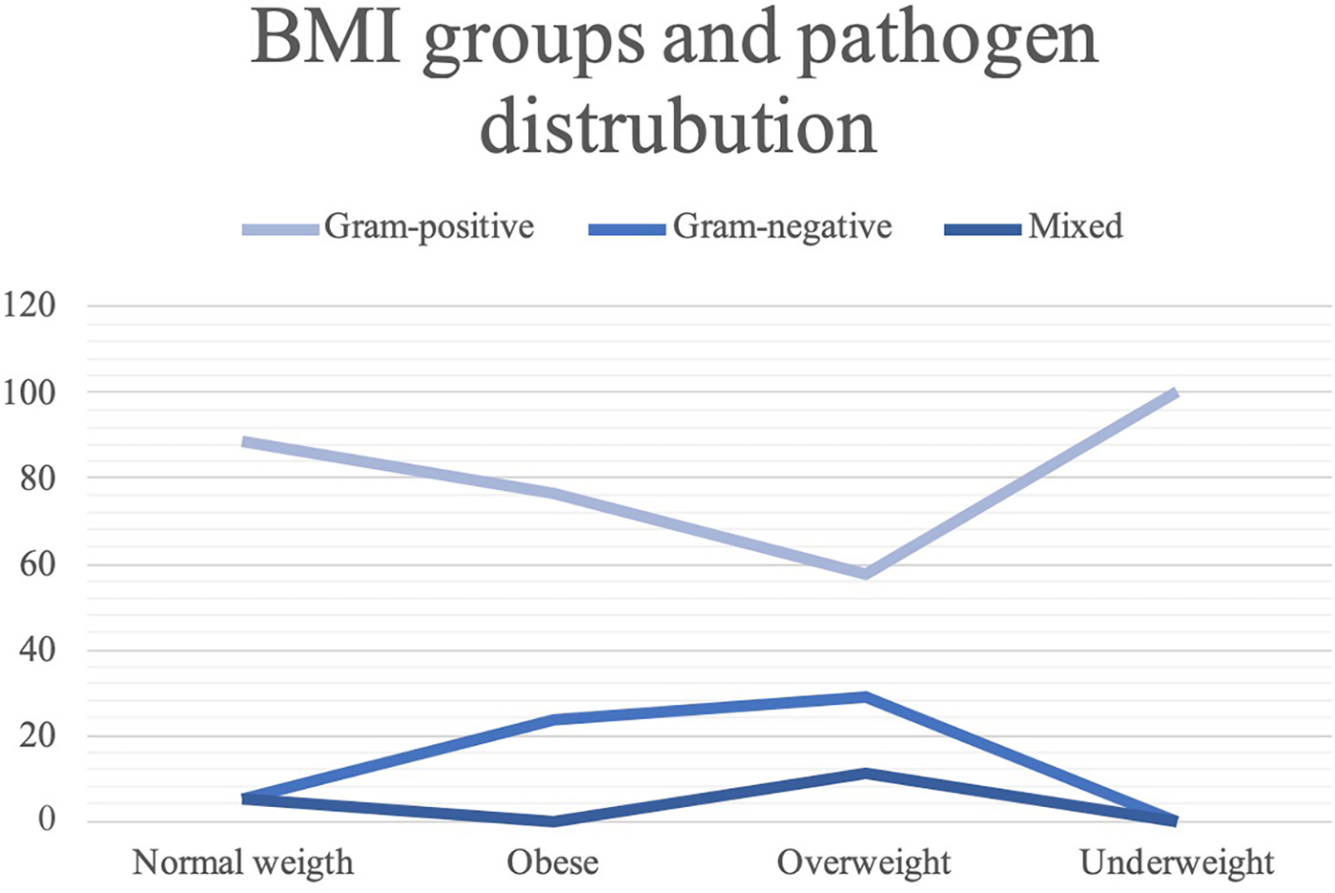
BMI groups and pathogen distribution.

We also analyzed the distribution of the different pathogens across patients’ demographics and past medical history. The findings from this study highlight the significant associations between certain patient characteristics and the type of pathogens found in infections. The significant correlation between BMI and pathogen class underscores the importance of considering BMI in managing and preventing infections (Figure 2). Normal-BMI patients showed a high prevalence of Gram-positive infections (88.46%), whereas obese and overweight patients had higher percentages of Gram-negative infections (23.53% and 28.89%, respectively).

Smoking status also revealed to be significantly associated to specific pathogens; particularly, non-smokers had a higher proportion of Gram-negative infections, while past smokers and active smokers exhibited higher percentages of mixed infections (8.11% and 12.90%, respectively).

Although radiotherapy and chemotherapy did not show significant associations, the type of reconstruction was an important factor. The higher incidence of mixed infections in patients undergoing two-stage reconstruction might be due to the prolonged exposure to potential pathogens during the reconstruction process or contamination during the period of expander inflation. The lack of significant association between drainage duration and pathogen type indicates that other factors may be more critical in determining the type of pathogens in surgical site infections. However, continuous monitoring and proper management of drainage systems remain essential to prevent infections.

According to the literature, breast infections typically manifest in a bimodal fashion. Early infections most commonly occur between the first and sixth week after surgery, presenting with both local and systemic signs such as fever, breast pain, erythema, and purulent fluid or drainage at the incision site. In contrast, late infections are less common and generally present with focal symptoms like dehiscence, drainage, cellulitis, and implant extrusion.

In this study, we investigated the timing of infection onset based on the causative pathogen. Our results indicate that the majority of infections present with an early onset (62.15%), occurring within 6 weeks of implant placement. Although no statistical difference was found in the distribution of pathogens based on the timing of onset, our findings suggest that Gram-negative infections presenting with a late onset are more likely to be caused by multi-resistant strains. Furthermore, our results indicate that Gram-negative bacteria are significantly more likely to present with only localised symptoms, rather than systemic symptoms. The differing presentations between pathogen groups could facilitate faster diagnosis and more appropriate antibiotic selection while awaiting culture results.

We also compared the distribution of Gram-positive and Gram-negative bacteria in relation to what we termed “clinical cure,” defined as the clinical absence of symptoms at 14 and 28 days from the onset of infection. Forty-eight patients underwent implant removal before reaching these time points.

Regarding the 14-day clinical cure, the majority of both Gram-positive and Gram- negative bacterial infections still exhibited local symptoms, whereas systemic symptoms were absent in 91.35% and 84.21% of cases, respectively.

By the 28-day clinical cure, systemic symptoms remained rare for both classes of pathogens, highlighting that systemic symptoms are very uncommon after 14 and 28 days from infection. However, local symptoms tended to be more common and prolonged. A higher percentage of Gram-positive bacterial infections showed local symptoms at day 28 compared to Gram-negative infections. Although this difference did not reach statistical significance, it suggests that Gram-positive bacteria may be associated with a longer duration of symptomatology.

The prevention of intraoperative contamination and post-operative infections is of utmost importance in breast reconstruction surgery. According to the guidelines for prevention of surgical site infection (SSI) provided by the Centers for Disease Control and Prevention (CDC), pre-operative antibiotic prophylaxis with a first-generation cephalosporin is recommended to reduce the risk of infection. Additionally, povidone- iodine irrigation during the operative phase is also considered effective in preventing SSI (11).

In our study, a crucial finding emerged from analysing the incidence of Staphylococcus aureus infections in patients who tested positive for the prophylactic MSSA/MRSA nasal swab. Specifically, 60% of patients with a positive nasal swab developed a Staphylococcus aureus infection, whereas only 23.42% of those with a negative swab result were subsequently infected by this pathogen. This result was statistically significant and underscores the critical role of preventive screening tests.

Given that *Staphylococcus aureus* is the most common pathogen causing breast implant infections, this finding highlights the necessity of implementing robust prophylactic measures against it. Patients who test positive for MSSA/MRSA nasal swabs should undergo decolonisation procedures. Effective protocols include daily lavages with 4% chlorhexidine, the application of 2% mupirocin nasal ointment starting 3–5 days before surgery, and the use of chlorhexidine mouthwash on the day of the surgery. These measures have been demonstrated to significantly reduce bacterial load and the risk of subsequent infection (12).

The results of our study have several important clinical implications for the management of periprosthetic infections in breast reconstruction surgeries. The predominance of Gram-positive bacteria, particularly Staphylococcus aureus, underscores the need for targeted antibiotic prophylaxis and treatment strategies against these pathogens (14). The presence of Gram-negative bacteria, along with the correlations we found between pathogen types and patient characteristics such as BMI, smoking status, reconstruction type, infection onset, and clinical manifestations, can guide the selection of the most appropriate antibiotics for individual patients based on these findings. Utilizing regional epidemiological data allows healthcare providers to tailor antibiotic regimens to the local microbiological landscape (5), potentially improving treatment outcomes and reducing the incidence of antibiotic resistance as well as the need for implant removal.

Limitations include the retrospective nature of the study, which relies on the accuracy of initial documentation. Additionally, the study involves data from multiple surgeons and institutions which can lead to individual variations among physicians, although there is a general homogeneity in infection management. Since the necessity of implant removal was a selection criterion, the isolated bacteria may not be representative of all types of infections but only most severe ones. Lastly, a larger sample size and inclusion of additional institutions may have revealed different pathogen frequencies not encountered in this patient cohort.

## Data Availability

The raw data supporting the conclusions of this article will be made available by the authors, without undue reservation.

## References

[1] 2022 ASPS procedural statistics release. (2023). Available online at: https://www.plasticsurgery.org/documents/News/Statistics/2022/plastic-surgery-statistics-report-2022.pdf (Accessed March 16, 2024). 10.1097/PRS.0000000000011235 38113105

[2] Broyles JM Balk EM Adam GP Cao W Bhuma MR Mehta S Implant-based versus autologous reconstruction after mastectomy for breast cancer: a systematic review and meta-analysis. Plast Reconstr Surg Glob Open. (2022) 10(3):e4180. 10.1097/GOX.0000000000004180 35291333 PMC8916208

[3] Lalani T. Breast implant infections: an update. Infect Dis Clin North Am. (2018) 32(4):877–84. 10.1016/j.idc.2018.06.007 30241714

[4] Yeo H Lee D Kim JS Eo PS Kim DK Lee JS Strategy for salvaging infected breast implants: lessons from the recovery of seven consecutive patients. Arch Plast Surg. (2021) 48(2):165–74. 10.5999/aps.2020.01578 33765733 PMC8007469

[5] Cohen JB Carroll C Tenenbaum MM Myckatyn TM. Breast implant-associated infections: the role of the national surgical quality improvement program and the local microbiome. Plast Reconstr Surg. (2015) 136(5):921–9. 10.1097/PRS.0000000000001682 26505698

[6] Rubino C Brongo S Pagliara D Cuomo R Abbinante G Campitiello N Infections in breast implants: a review with a focus on developing countries. J Infect Dev Ctries. (2014) 8(9):1089– 95. 10.3855/jidc.3898 25212072

[7] Ozturk C Ozturk CN Platek M Soucise A Laub P Morin N Management of expander- and implant-associated infections in breast reconstruction. Aesthetic Plast Surg. (2020) 44(6):2075–82. 10.1007/s00266-020-01923-8 32840671

[8] Piper ML Roussel LO Koltz PF Wang F Singh K Chin R Characterizing infections in prosthetic breast reconstruction: a validity assessment of national health databases. J Plast Reconstr Aesthet Surg. (2017) 70(10):1345–53. 10.1016/j.bjps.2017.05.004 28619483

[9] Banuelos J Abu-Ghname A Asaad M Vyas K Sohail MR Sharaf B. Microbiology of implant-based breast reconstruction infections: a systematic review. Ann Plast Surg. (2020) 85(2):194–201. 10.1097/SAP.0000000000001974 31513083

[10] Al-Halabi B Viezel-Mathieu A Shulman Z Behr MA Fouda Neel O. Breast implant mycobacterial infections: an epidemiologic review and outcome analysis. Plast Reconstr Surg. (2018) 142(5):639e–52. 10.1097/PRS.0000000000004892 30096121

[11] Huang N Liu M Yu P Wu J. Antibiotic prophylaxis in prosthesis-based mammoplasty: a systematic review. Int J Surg. (2015) 15:31–7. 10.1016/j.ijsu.2015.01.020 25638736

[12] Mangram AJ Horan TC Pearson ML Silver LC Jarvis WR. Guideline for prevention of surgical site infection, 1999. Hospital infection control practices advisory committee. Infect Control Hosp Epidemiol. (1999) 20(4):250–80. 10.1086/501620 10219875

[13] Weichman KE Levine SM Wilson SC Choi M Karp NS. Antibiotic selection for the treatment of infectious complications of implant-based breast reconstruction. Plast Surg. (2013) 71(2):140–3. 10.1097/SAP.0b013e3182590924 23486147

[14] Nickel KB Fox IK Margenthaler JA Wallace AE Fraser VJ Olsen MA. Effect of noninfectious wound complications after mastectomy on subsequent surgical procedures and early implant loss. J Am Coll Surg. (2016) 222(5):844–52.e1. 10.1016/j.jamcollsurg.2016.01.050 27010582 PMC4846523

